# Trends of Randomized Clinical Trials Citing Prior Systematic Reviews, 2007-2021

**DOI:** 10.1001/jamanetworkopen.2023.4219

**Published:** 2023-03-23

**Authors:** Yuanxi Jia, Bingli Li, Zhirong Yang, Fuxiao Li, Ziyi Zhao, Chang Wei, Xuhao Yang, Qianyi Jin, Di Liu, Xin Wei, Jennifer Yost, Hans Lund, Jinling Tang, Karen A. Robinson

**Affiliations:** 1 Shenzhen Institute of Advanced Technology, Chinese Academy of Sciences, Shenzhen, China; 2 Department of Public Health and Primary Care, School of Clinical Medicine, University of Cambridge, Cambridge, United Kingdom; 3 Center for Diversity in Public Health Leadership Training, Kennedy Krieger Institute, Johns Hopkins University, Baltimore, Maryland; 4 Department of Cardiology, Virginia Commonwealth University, Richmond, Virginia; 5 M. Louise Fitzpatrick College of Nursing, Villanova University, Villanova, Pennsylvania; 6 Section Evidence-Based Practice, Western Norway University of Applied Sciences, Bergen, Norway; 7 School of Public Health and Primary Care, Chinese University of Hong Kong, Hong Kong; 8 Department of Medicine, School of Medicine, Johns Hopkins University, Baltimore, Maryland

## Abstract

**Importance:**

Systematic reviews can help to justify a new randomized clinical trial (RCT), inform its design, and interpret its results in the context of prior evidence.

**Objective:**

To assess trends and factors associated with citing (a marker of the use of) prior systematic reviews in RCT reports.

**Design, Setting, and Participants:**

This cross-sectional study investigated 737 Cochrane reviews assessing health interventions to identify 4003 eligible RCTs, defined as those included in an updated version but not in the first version of a Cochrane review and published 2 years after the first version of the Cochrane review was published.

**Main Outcomes and Measures:**

The primary outcome was the citation of prior systematic reviews, Cochrane or others, as determined by screening references of eligible RCTs. Factors that may be associated with the citation of prior systematic reviews were also examined.

**Results:**

Among 4003 eligible RCTs, 1241 studies (31.0%) cited Cochrane reviews, 1698 studies (42.4%) cited prior non-Cochrane reviews, and 2265 studies (56.6%) cited either type of systematic review or both; 1738 RCTs (43.4%) cited no systematic reviews. The percentage of RCTs citing prior Cochrane reviews, non-Cochrane reviews, and either or both types of review increased from 28 studies (15.3%), 46 studies (25.1%), and 65 studies (35.5%) of 183 RCTs before 2008 to 42 studies (40.8%), 65 studies (64.1%), and 73 studies (71.8%) of 102 RCTs since 2020, respectively; the annual increases were 1.9% (95% CI, 1.4%-2.3%), 3.3% (95% CI, 2.9%-3.7%), and 3.0% (95% CI, 2.5%-3.5%), respectively. The proportion of RCTs citating prior systematic reviews varied considerably across clinical specialties, ranging from 28 of 106 RCTs (26.4%) in ophthalmology to 386 of 553 RCTs (69.8%) in psychiatry (*P* < .001). RCTs with 100 participants or more (risk ratio [RR], 1.16; 95% CI, 1.03-1.30), nonindustry funding (RR, 1.43; 95% CI, 1.27-1.61), and authors from high-income countries (RR, 1.10; 95% CI, 1.03-1.17) were more likely to cite systematic reviews than those with fewer than 100 participants, industry funding, and authors from low- and middle-income countries, respectively. A journal requirement to cite systematic reviews was not associated with the likelihood of citing a systematic review.

**Conclusions and Relevance:**

This study found that the citation of prior systematic reviews in RCT reports improved over time, but approximately 40% of RCTs failed to do so. These findings suggest that reference to prior evidence for initiating, designing, and reporting RCTs should be further emphasized to assure clinical relevance, improve methodological quality, and facilitate interpretation of new results.

## Key Points

**Question:**

Has the citation of prior systematic reviews in reports of randomized clinical trials improved over time?

**Findings:**

In this cross-sectional study of 4003 randomized clinical trials (RCTs), the percentage of RCTs citing systematic reviews increased from 35.5% in 2007 to 2008 to 71.8% since 2020, with an annual rate of increase of 3.0%. RCTs with 100 participants or more, nonindustry funders, and authors from high-income countries were more likely to cite systematic reviews than those with fewer than 100 participants, industry funders, and authors from low- and middle-income countries.

**Meaning:**

These findings suggest that the citation of prior systematic reviews in reports of RCTs has improved over time but may need further improvement.

## Teaser

This cross-sectional study investigates trends in the citation of Cochrane reviews and other systematic reviews by randomized clinical trials from 2007 to 2021.

## Introduction

Randomized clinical trials (RCTs) should be justified, designed, and interpreted in the context of prior evidence.^1,2,3,4^ Failure to consider prior evidence may be associated with low clinical relevance, compromised methodological quality, and even redundant RCTs on clinical questions for which sufficient quality evidence is already available. Such studies may waste valuable resources, unnecessarily put patients at potential harm, and damage public trust in scientific research.^5,6,7,8,9,10^

According to the *Cochrane Handbook for Systematic Reviews of Interventions*, a systematic review “attempts to collate all empirical evidence that fits prespecified eligibility criteria in order to answer a specific research question” and is characterized by a clear set of objectives, a reproducible methodology, a comprehensive search, an assessment of the validity of findings, and a systematic presentation.^11^ A high-quality and up-to-date systematic review comprehensively synthesizes prior evidence and can help to ensure that new RCTs are worthwhile and informative.^1,2,3,4^ For example, systematic reviews may help justify the necessity of a new trial, plan its sample size, and overcome major methodological problems of prior similar trials. When the new trial is completed, systematic reviews can help assess its outcomes among the overall evidence by comparing and synthesizing its findings with those of prior similar trials.

Making reference to or citing a relevant systematic review in an RCT report may be used as an indication of the use of prior evidence. In the past 2 decades, many stakeholders have endorsed the importance of and made great efforts to promote the citation of prior systematic reviews in RCT reports. For example, the Consolidated Standards of Reporting Trials (CONSORT) reporting guideline statement recommends that an RCT report include “a reference to a systematic review of previous similar trials or a note of the absence of such trials.”^12^ In 2017, an international group of health research funders called for funding only research that had been set in the context of existing systematic reviews or had robustly demonstrated a research gap.^13^ Further, some journals have explicitly required citation of prior relevant systematic reviews since 2010.^14^ In addition, researchers have been advocating citing systematic reviews, including through international organizations, such as the Evidence-Based Research Network^15^ and EVBRES (Evidence-Based Research) a Cost Action.^1,2,3,16^

A 2022 study^17^ suggested that researchers frequently failed to cite a relevant systematic review. However, it remains unclear whether the reference to prior evidence has improved in RCTs and what factors may be associated with this practice.^17^ We thus conducted this study to address these research questions. Objectives were to assess the citation of systematic reviews in RCT reports, compare the citation of systematic reviews in RCT reports across clinical specialties, and assess the factors associated with citing systematic reviews in RCT reports.

## Methods

In this cross-sectional study, we identified eligible RCTs from systematic reviews conducted by Cochrane (referred to as Cochrane reviews). The citation of prior systematic reviews was assessed in reports of these RCTs. All data in this study were obtained from open sources. Therefore, ethics review and informed consent exemptions were approved by the local Institutional Review Board of the Shenzhen Institute of Advanced Technology, Chinese Academy of Sciences. We followed the Strengthening the Reporting of Observational Studies in Epidemiology (STROBE) reporting guideline in reporting this study.

### Selection of Eligible Cochrane Reviews

All Cochrane reviews indexed in the Cochrane Database of Systematic Reviews up to October 2021 were screened. A Cochrane review was considered eligible if it included only RCTs or quasi-RCTs; it assessed the effectiveness, efficacy, or safety of a health intervention; it had been updated at least once (we included the first and latest versions); none of its versions had been withdrawn; and it included at least 1 meta-analysis to ensure similarity of RCTs. We used the Web of Science to assess the citation of systematic reviews by eligible RCTs. Therefore, we further excluded Cochrane reviews that were not indexed in the Web of Science.

### Selection of Eligible RCTs

An RCT cannot cite a prior systematic review if no prior systematic review is available. Therefore, we used a special design to include RCTs with at least 1 Cochrane review available to cite to reduce selection bias. Eligible RCTs were selected by comparing the first and latest version of an eligible Cochrane review. Specifically, an eligible RCT was included in the latest version of an eligible Cochrane review, not included in the first version of this Cochrane review, and published at least 2 years later than the first version of this Cochrane review was published. This 2-year grace period accounted for publication delays and ensured that the first version of the Cochrane review was available to cite when RCT reports were under development (eFigure in Supplement 1). Therefore, eligible RCTs could cite the first version of the Cochrane review.

In addition, we considered only RCT reports published in English journals as full articles. When multiple reports of an RCT were available, we considered only the primary report as decided by the authors of the Cochrane review. Eligible RCTs were identified by comparing references under the “References to Included Studies” section between the first and the latest version of an eligible Cochrane review. An RCT was counted only once if included in multiple Cochrane reviews.

### Citation of Systematic Reviews

Trialists may cite systematic reviews for various purposes, such as to justify a new RCT, inform its design, or put its findings in the context of prior similar RCTs. In this study, we considered only whether a prior systematic review was cited, regardless of the purposes.

#### Citation of Cochrane Reviews

A Cochrane review may be updated multiple times. Citing any prior version of the Cochrane review by the eligible RCT was considered a citation.

#### Citation of Non-Cochrane Reviews

Eligible RCTs may cite prior, relevant non-Cochrane reviews, that is, systematic reviews conducted independently of Cochrane. Operationally, a non-Cochrane review was screened to confirm that it met the following methodological prerequisites: at least 2 bibliographic databases were searched; any form of quality or risk of bias assessment using validated tools or self-developed criteria was reported in the results section; and the review intended to include RCTs. We included non-Cochrane reviews that searched for RCTs but identified none and excluded those that considered only observational studies. We also included non-Cochrane reviews if the result of quality or risk of bias assessment was reported as a supplement with an explicit link in the results section. To be clinically relevant, a non-Cochrane review was expected to assess the efficacy, effectiveness, or safety of health interventions similar to eligible RCTs and assess health conditions similar to RCTs.

The title and abstract of references cited by each eligible RCT were obtained from the Web of Science. Then, titles of these references were screened for review or meta-analysis and abstracts were screened for PubMed, Medline, Embase, Cochrane, or Web of Science. Full texts of references with the keywords mentioned previously were obtained and manually screened by 2 epidemiologists (Y.J., Z.Y., F.L., and D.L.) for methodological prerequisites and 2 clinicians (including X.W.) for clinical relevancy. Systematic reviews published in journals other than the *Cochrane Database of Systematic Reviews* were considered Cochrane reviews if they were reprints of or directly derived from a Cochrane review.

### Data Abstraction

The following data items were abstracted from eligible RCTs: year published, sample size, number of recruiting centers, funders, country of first and senior author affiliations, and journals where published. When co–first authors were reported, we considered the first 1. When co–senior authors were reported, we considered the last 1. When multiple affiliations were reported for the same author, the country of the first affiliation was considered.

Author guidelines of journals where eligible RCTs were published were reviewed for requirements about citation of systematic reviews in RCTs. The requirement was classified into 1 of 3 categories: explicit requirement on citing a systematic review, referring to the CONSORT statement, and no requirement. The data abstraction was conducted by 2 authors independently to reduce information bias, and a third researcher resolved any disagreements (Y.J., Z.Z., C.W., Q.J., and X.Y.).

### Statistical Analysis

We calculated the percentage of eligible RCTs citing systematic reviews by type (Cochrane review, non-Cochrane review, or either), year of publication, and clinical specialty. Clinical specialties included obstetrics and gynecology, psychiatry, pediatrics, neurology, urology, oncology, respiratory disorders, cardiovascular disorders, orthopedics, anesthesiology, dentistry, dermatology, ophthalmology, gastroenterology and hepatology, endocrinology, and others. In the analysis, specialties with fewer than 100 eligible RCTs were classified into the category of others. A Cochran-Mantel-Haenszel test was used to compare the citation of systematic reviews across clinical specialties.

The trend of eligible RCTs citing Cochrane reviews, non-Cochrane reviews, or either type of systematic review was assessed by linear regression models. A log-binomial model was used to control for confounding to assess the association between the citation of systematic reviews and characteristics of eligible RCTs. Factors included sample size (<100 vs ≥100 individuals),^18,19,20,21,22^ number of recruiting centers (single center vs multicenter), funding (industry funded, not industry funded, no funding, or not reported), country of authors (high-income countries vs low- and middle-income countries), the requirement of journals (explicit requirement, referring to the CONSORT statement, or no requirement), year of publication, and clinical specialty. We considered an RCT to be conducted by researchers from high-income countries if its first author or senior author was affiliated with an organization in a high-income country.^23^ No missing values existed for any variables extracted. Adjusted relative differences with 95% CIs were estimated. SAS statistical software version 9.4 (SAS Institute) was used for data cleaning and analysis. All statistical analyses were 2-sided based on *P* values, and the level of statistical significance was set at .05. Data analysis was conducted between February and May 2022.

## Results

**Figure 1.**
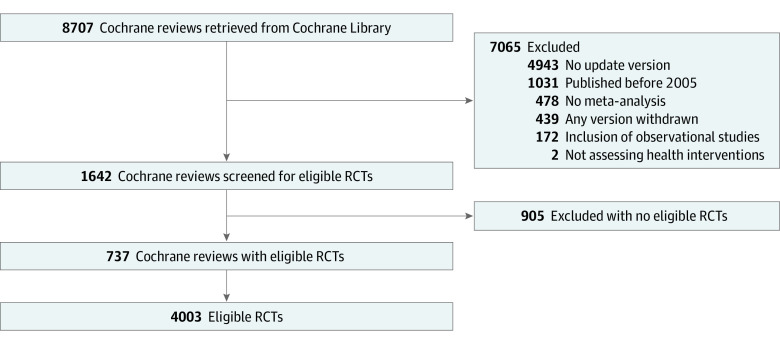
Selection of Eligible Randomized Clinical Trials (RCTs) The process to select eligible RCTs is shown.

A total of 8707 Cochrane reviews were identified and screened, of which 1642 reviews were considered eligible and searched for eligible RCTs. Eventually, 4003 eligible RCTs published in 1205 journals were identified from 737 Cochrane reviews (Figure 1). No eligible RCTs were excluded from analyses due to missing values.

### Selection of Eligible RCTs

Characteristics of eligible RCTs are shown in Table 1. Most eligible RCTs recruited at least 100 participants (2298 RCTs [57.4%]), recruited participants from a single center (2320 RCTs [58.0%]), had first or senior authors from high-income countries (2758 RCTs [68.9%]), were published in journals referring to the CONSORT statement (2515 RCTs [62.8%]), and were supported by nonindustry funders (1998 RCTs [49.9%]). Because too few eligible RCTs were published in 2007 and 2021, they were combined with those published in 2008 and 2020, respectively, in analyses.

**Table 1.** Characteristics of Eligible RCTs

Category	Eligible RCTs, No. (%)			Total
	Citing Cochrane reviews	Citing non-Cochrane reviews	Citing either type of systematic review	
Total	1241 (31.0)	1698 (42.4)	2265 (56.6)	4003
Sample size, No. individuals				
<100	472 (27.7)	658 (38.6)	880 (51.6)	1705
≥100	769 (33.5)	1040 (45.3)	1385 (60.3)	2298
No. of centers				
Single center	706 (30.4)	976 (42.1)	1294 (55.8)	2320
Multicenter	535 (31.8)	722 (42.9)	971 (57.7)	1683
Country of authors				
Low- and middle-income countries	345 (27.7)	499 (40.1)	660 (53.0)	1245
High-income countries	896 (32.5)	1199 (43.5)	1605 (58.2)	2758
Requirement of journals				
Not requiring citation of systematic reviews	414 (30.8)	570 (42.3)	754 (56.0)	1346
Referring to the CONSORT statement	776 (30.9)	1065 (42.3)	1424 (56.6)	2515
Explicitly requiring citation of systematic reviews	51 (35.9)	63 (44.4)	87 (61.3)	142
Funding source				
Industry	160 (21.7)	246 (33.4)	330 (44.8)	737
Nonindustry	731 (36.6)	966 (48.3)	1279 (64.0)	1998
No external funding	82 (28.5)	126 (43.8)	158 (54.9)	288
Not reported	268 (27.3)	360 (36.7)	498 (50.8)	980
Year of publication				
2007	8 (13.1)	14 (23.0)	19 (31.1)	61
2008	20 (16.4)	32 (26.2)	46 (37.7)	122
2009	39 (19.8)	54 (27.4)	74 (37.6)	197
2010	71 (27.3)	79 (30.4)	124 (47.7)	260
2011	90 (25.6)	121 (34.5)	179 (51.0)	351
2012	114 (28.6)	149 (37.3)	213 (53.4)	399
2013	131 (31.1)	171 (40.6)	239 (56.8)	421
2014	144 (30.4)	194 (40.9)	254 (53.6)	474
2015	155 (32.2)	206 (42.7)	282 (58.5)	482
2016	145 (37.8)	195 (50.8)	248 (64.6)	384
2017	123 (37.2)	166 (50.2)	217 (65.6)	331
2018	98 (39.2)	144 (57.6)	176 (70.4)	250
2019	61 (36.3)	107 (63.7)	120 (71.4)	168
2020	41 (45.1)	56 (61.5)	64 (70.3)	91
2021	1 (9.1)	9 (81.8)	9 (81.8)	11

Abbreviations: CONSORT, Consolidated Standards of Reporting Trials; RCT, randomized clinical trial.

We extracted 112 801 references from 3882 eligible RCTs using the Web of Science; of these, 10 457 references were identified as possible systematic reviews using keywords. From this methodological screening, we obtained 3074 systematic reviews, of which 209 reviews were clinically irrelevant and subsequently excluded. Eventually, 2865 references were considered non-Cochrane reviews.

We could not extract references from 121 eligible RCTs using the Web of Science. References of those RCTs were manually searched for non-Cochrane reviews. A total of 71 references were considered non-Cochrane reviews.

### Trends of Citing Systematic Reviews

**Figure 2.**
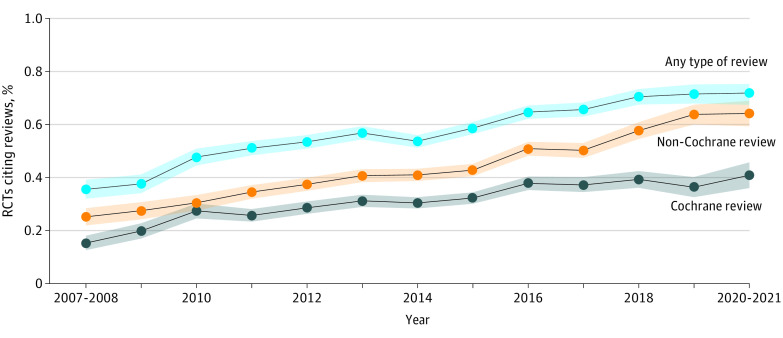
Trends of Eligible Randomized Clinical Trials (RCTs) Citing Systematic Reviews Lines indicate point estimates; shaded areas, 95% CIs.

Overall, 1241 eligible RCTs (31.0%) cited Cochrane reviews, 1698 RCTs (42.4%) cited non-Cochrane reviews, and 2265 RCTs (56.6%) cited either type of systematic review. Thus, 1738 RCTs (43.4%) failed to cite systematic reviews. Table 1 and Figure 2 show the trends of citing systematic reviews in eligible RCTs.

The percentage of eligible RCTs citing Cochrane reviews increased from 28 of 183 RCTs (15.3%) in 2007 to 2008 to 42 of 102 RCTs (40.8%) in 2020 to 2021, with an annual increase of 1.9% (95% CI, 1.4%-2.3%). The percentage of eligible RCTs citing non-Cochrane reviews increased from 46 of 183 RCTs (25.1%) in 2007 to 2008 to 65 of 102 RCTs (64.1%) in 2020 to 2021, with an annual increase of 3.3% (95% CI, 2.9%-3.7%). The percentage of eligible RCTs citing either type of systematic review increased from 65 of 183 RCTs (35.5%) in 2007 to 2008 to 73 of 102 RCTs (71.8%) in 2020 to 2021, with an annual increase of 3.0% (95% CI, 2.5%-3.5%). Overall, the percentage of eligible RCTs citing Cochrane reviews was 12.6% (95% CI, 8.6%-16.7%) lower than that of RCTs citing non-Cochrane reviews.

### Trends of Eligible RCTs Citing Systematic Reviews

As shown in Table 2, the percentage of eligible RCTs citing systematic reviews varied considerably by clinical specialty (*χ^2^* = 402; *P* < .001). For citing Cochrane reviews, the percentage was highest in eligible RCTs in psychiatry (233 of 553 RCTs [42.1%]) and lowest in dermatology (10 of 109 RCTs [9.2%]). For citing non-Cochrane reviews, the highest and the lowest percentages were found in RCTs in orthopedics (108 of 193 RCTs [56.0%]) and ophthalmology (16 of 106 RCTs [15.1%]), respectively. For citing either type of systematic review, the highest citation percentage was in RCTs in psychiatry (386 RCTs [69.8%]) and the lowest percentage in ophthalmology (28 RCTs [26.4%]).

**Table 2.** Eligible RCTs Citing Systematic Reviews by Clinical Specialty

Clinical specialty	Eligible RCTs, No.	Eligible RCTs citing systematic reviews, No. (%)		
		Cochrane reviews	Non-Cochrane reviews	Either type of review
Obstetrics and gynecology	557	217 (39.0)	241 (43.3)	342 (61.4)
Psychiatry	553	233 (42.1)	291 (52.6)	386 (69.8)
Pediatrics	452	162 (35.8)	203 (44.9)	272 (60.2)
Neurology	395	141 (35.7)	219 (55.4)	259 (65.6)
Urology	318	65 (20.4)	102 (32.1)	142 (44.7)
Oncology	232	46 (19.8)	71 (30.6)	95 (40.9)
Respiratory disorders	215	76 (35.3)	83 (38.6)	127 (59.1)
Cardiovascular disorders	214	56 (26.2)	103 (48.1)	123 (57.5)
Orthopedics	193	61 (31.6)	108 (56)	127 (65.8)
Anesthesiology	154	46 (29.9)	58 (37.7)	90 (58.4)
Dentistry	118	18 (15.3)	57 (48.3)	63 (53.4)
Dermatology	109	10 (9.2)	24 (22.0)	31 (28.4)
Ophthalmology	106	15 (14.2)	16 (15.1)	28 (26.4)
Other	387	95 (24.5)	122 (31.5)	180 (46.5)

Abbreviation: RCT, randomized clinical trial.

### Factors Associated With Citing Either Type of Systematic Review

There were 3 factors associated with citing either type of systematic review. RCTs that recruited at least 100 participants were more likely (risk ratio [RR], 1.16; 95% CI, 1.03-1.30) to cite systematic reviews than those that recruited fewer than 100 participants. RCTs that were supported by nonindustry funders (RR, 1.43; 95% CI, 1.27-1.61), received no external funding (RR, 1.27; 95% CI, 1.08-1.50), and did not report funding sources (RR, 1.26; 95% CI, 1.08-1.46) were more likely to cite either type of systematic review than those supported by industry funders. RCTs with first or senior authors from high-income countries were more likely (RR, 1.10; 95% CI, 1.03-1.17) to cite systematic reviews than RCTs with first and senior authors from low- and middle-income countries. The number of recruiting centers and the requirement of journals were not associated with the citation of systematic reviews (eTable in Supplement 1).

## Discussion

A systematic review of prior research can help identify research gaps and ensure that new RCTs do not reexamine questions that have already been addressed.^24,25,26^ In addition, a systematic review that critically assesses the quality of prior similar studies may provide details that improve the design of new RCTs. When new RCTs are reported, systematic reviews may be used to explicitly integrate results of new RCTs into the existing evidence base. Although this cross-sectional study found that the percentage of RCTs citing systematic reviews improved from 35.5% in 2007 to 2008 to 71.8% since 2020, more than a quarter of RCTs still failed to do so.

Approximately 56.6% of RCTs included in our study cited systematic reviews, an estimate similar to prior estimates, which were generally greater than 60%.^27,28,29^ The slight difference may be associated with methodological differences. For example, prior estimates were generated from a single cohort of RCTs sharing similar health conditions and interventions under investigation, while our study sample included RCTs addressing various questions. In addition, the citation of systematic reviews improved in the last decade; therefore, estimates may be associated with the time when the RCTs were published.

RCTs with industry funding were less likely to cite systematic reviews. This is not surprising given that prior research has found that industry-funded RCTs were less likely to follow guidelines or recommendations on the design, conduct, or report of RCTs. For example, industry-funded RCTs were less likely to report results on trial registries^30^ and more likely to report biased results in favor of sponsor products.^31^ Although we expected that RCTs published in journals requiring citation of systematic reviews would be more likely to cite reviews, results did not support this assumption. This may be associated with a lack of enforcement of such requirements. Some reviews labeled as systematic reviews were not considered reviews per our criteria because of the failure to conduct a comprehensive search or quality assessment.^32,33^ In addition, the citation of systematic reviews varied considerably by clinical specialty and thus should be especially prompted in clinical specialties with lower citation rates.

We hypothesized that RCTs were more likely to cite Cochrane reviews because the design of the study ensured the availability of at least 1 Cochrane review to cite, and the quality of Cochrane reviews is generally higher than that of non-Cochrane reviews.^34^ However, the citation of Cochrane reviews was significantly lower than that of non-Cochrane reviews, especially in recent years. There may be steps that Cochrane can take to improve the citation of Cochrane reviews. For example, the term systematic review may be added to the title of Cochrane reviews to be more easily identified by researchers who search PubMed or Embase for systematic reviews rather than the Cochrane Library. In addition, stakeholders may consider transitioning all Cochrane reviews to open access.^35^

Several obstacles may hinder the citation of systematic reviews. For example, high-quality and up-to-date systematic reviews are not always available to justify a new RCT and guide its design. We based our study sample on existing Cochrane reviews, but in many fields, the evidence has not been synthesized by existing systematic reviews to date.^36^ Although we considered only non-Cochrane reviews that met some limited quality criteria, researchers should be aware that the quality of many existing systematic reviews is suboptimal.^37,38,39^ To increase the citation of prior systematic reviews, more resources and researchers with expertise and experience are needed to improve the coverage of health topics by high-quality and up-to-date systematic reviews.^40,41^ In addition to researchers and journals, more stakeholders in the research system, including academic institutions, institutional review boards, and funding agencies,^42^ may play a role by mandating a systematic review of prior research as a prerequisite to approving new applications.

### Limitations

There are several limitations of our study. First, we considered reports of only RCTs and systematic reviews indexed in the Web of Science. Thus, the accuracy of our estimates depended on the coverage of journals and quality of indexing on the Web of Science. It was challenging to estimate the magnitude and direction of potential biases affecting estimates. Second, all eligible RCTs were identified from Cochrane reviews. It is unclear to what extent these RCTs could represent all published RCTs, although reviews covered topics across 15 specialties. Third, we used keywords to screen for possible systematic reviews from the reference list of eligible RCTs. There may be systematic reviews cited that we failed to identify. Fourth, although we developed criteria to define non-Cochrane systematic reviews, including a comprehensive search in 2 bibliographic databases and quality assessment of included RCTs, we did not formally assess the quality of these reviews. The quality of some non-Cochrane reviews may have been too low to guide the design of new RCTs. However, because we applied the same criteria to all non-Cochrane reviews, the trend of RCTs citing prior systematic reviews should not be significantly biased. Fifth, although the quality of Cochrane reviews is generally better than that of non-Cochrane reviews,^43^ there is evidence showing that the quality of some Cochrane reviews is also suboptimal.^34^ However, these low-quality Cochrane reviews are unlikely to be associated with estimates of RCTs citing prior systematic reviews.

Citing prior systematic reviews may imply that authors were more likely to consider prior evidence, and thus such citation was used as a proxy for considering prior evidence in this study. However, we should be aware that authors may cite a systematic review for many reasons; citing prior systematic reviews is not a direct measure of considering prior evidence. The agreement between citing prior systematic reviews and considering prior evidence remains unclear.

## Conclusions

This cross-sectional study found that the citation of prior systematic reviews in reports of RCTs improved over time, but overall, approximately 40% of reports of RCTs failed to do so. These findings suggest that evidence-based research, such as through the use of a systematic review, should be promoted to inform the justification and design of new RCTs and to report their findings within the context of what is already known.

## Notes

Supplement 1. **eFigure.** Defining Eligible Randomized Clinical Trials **eTable.** Factors Associated With Citation of Systematic Reviews

Supplement 2.
Data Sharing Statement
