# Supplementary material for: Trends of Randomized Clinical Trials Citing Prior Systematic Reviews, 2007-2021
Source: JAMA Netw Open. 2023 Mar 23;6(3):e234219. doi: 10.1001/jamanetworkopen.2023.4219 (PMC10037150; doi:10.1001/jamanetworkopen.2023.4219)
Supplement: Supplement 1. — eFigure. Defining Eligible Randomized Clinical Trials eTable. Factors Associated With Citation of Systematic Reviews [file jamanetwopen-e234219-s001.pdf]

Supplemental Online Content

Jia Y, Li B, Yang Z, et al. Trends of randomized clinical trials citing prior systematic reviews, 2007-2021. *JAMA Netw Open*. 2023;6(3):e234219. doi:10.1001/jamanetworkopen.2023.4219

eFigure. Defining Eligible Randomized Clinical Trials

eTable. Factors Associated With Citation of Systematic Reviews

This supplemental material has been provided by the authors to give readers additional information about their work.

© 2023 Jia Y et al. *JAMA Network Open.*

eFigure. Defining Eligible Randomized Clinical Trials

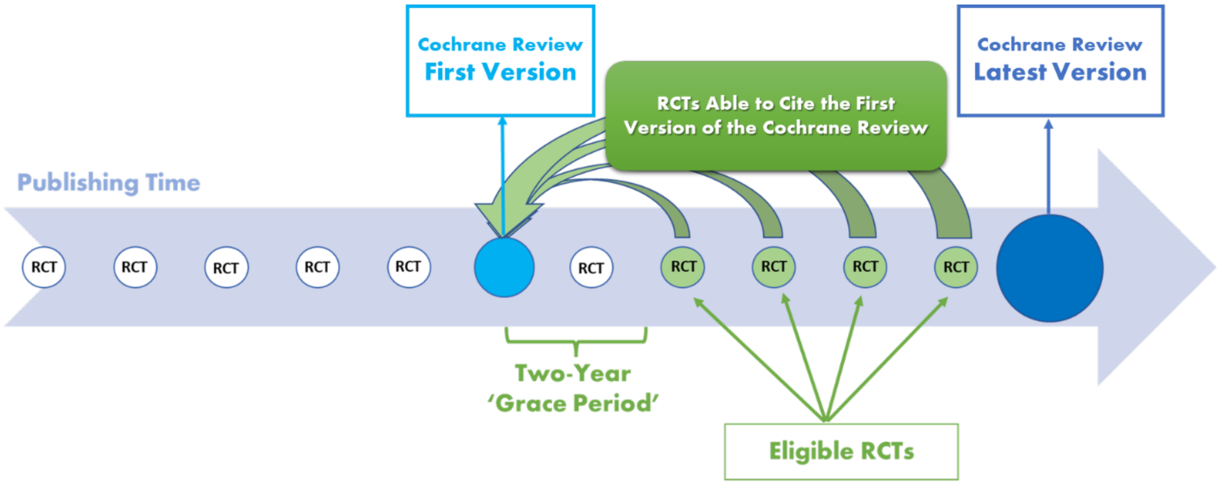

© 2023 Jia Y et al. *JAMA Network Open.*

eTable. Factors Associated With Citation of Systematic Reviews

Factor*	Risk Ratio (95%CI)
Sample Size	
≥100 vs. <100	1.15 (95%CI: 1.03-1.29)**
No. of Recruiting Centers	
Multi-Center vs. Single Center	0.98 (95%CI: 0.92-1.05)
Funding Source	
Non-Industry vs. Industry	1.43 (95%CI: 1.29-1.58)**
No Funding vs. Industry	1.27 (95%CI: 1.10-1.45)**
Not Reported vs. Industry	1.26 (95%CI: 1.10-1.44)**
Country	
Developed vs. Developing	1.10 (95%CI: 1.01-1.19)**
Requirement of Journal	
Explicitly Requiring Citation of Systematic Reviews vs. Not	0.99 (95%CI: 0.93-1.04)
Referring to the CONSORT Statement vs. Not	0.98 (95%CI: 0.85-1.14)

*Adjusted for the year of publishing and health conditions.
**Significant association.

© 2023 Jia Y et al. *JAMA Network Open.*
