# Supplementary material for: Trends of Randomized Clinical Trials Citing Prior Systematic Reviews, 2007-2021
Source: JAMA Netw Open. 2023 Mar 23;6(3):e234219. doi: 10.1001/jamanetworkopen.2023.4219 (PMC10037150; doi:10.1001/jamanetworkopen.2023.4219)
Supplement: Supplement 2. — Data Sharing Statement [file jamanetwopen-e234219-s002.pdf]

Data Sharing Statement

Jia. Trends of randomized clinical trials citing prior systematic reviews, 2007-2021. *JAMA Netw Open*. Published online March 23, 2023. doi:10.1001/jamanetworkopen.2023.4219

Data
Data available: Yes
Data types: Data (not involving human participants), Data dictionary
How to access data: Data will be available by emailing Yuanxi Jia at yjia12jhu@gmail.com **When available:** With publication

Supporting Documents
Document types: None

Additional Information
Who can access the data: Data will be available by emailing Yuanxi Jia at yjia12jhu@gmail.com
Types of analyses: All analyses
Mechanisms of data availability: with investigator support

© 2023 Jia Y et al. *JAMA Network Open*.
